# Effective Use of Novel Auxotrophic *Agrobacterium tumefaciens* Strains for Transformation and Biocontainment

**DOI:** 10.3390/plants14060925

**Published:** 2025-03-15

**Authors:** Lichi Zhong, Huiting Guo, Ling Wu, Qiang Cheng

**Affiliations:** State Key Laboratory of Tree Genetics and Breeding, Co-Innovation Center for Sustainable Forestry in Southern China, Nanjing Forestry University, Nanjing 210037, China; zlc572040483@163.com (L.Z.); htinguo125@163.com (H.G.); wuling@njfu.edu.cn (L.W.)

**Keywords:** *Agrobacterium tumefaciens*, auxotrophic strains, *Nicotiana benthamiana*, *Agrobacterium* overgrowth, biocontainment

## Abstract

Auxotrophic strains of *Agrobacterium tumefaciens* have been developed to address the *Agrobacterium* overgrowth issue in plant genetic transformation; however, their application remains limited. Here, we generated novel histidine and leucine auxotrophic strains of *A. tumefaciens* EHA105, namely EHA105hisD− and EHA105leuA−, as well as a dual auxotrophic strain EHA105hisD−leuA−, through gene deletion. The transient expression efficiency and survival rate of these three auxotrophic strains in *Nicotiana benthamiana* were significantly impaired but could be restored to wild-type EHA105 levels by supplementation with appropriate concentrations of the corresponding amino acids (CAAs). The use of these three auxotrophic strains for the genetic transformation of *N. benthamiana* resulted in a significant reduction in *Agrobacterium* overgrowth and achieved transformation efficiency comparable to wild-type EHA105, when appropriate exogenous concentrations of the CAAs were supplied during the co-cultivation stage. Furthermore, through incubation experiments on various plants and soil, it was confirmed that the incidence of surviving cells from these three auxotrophic strains was much lower than that observed for the wild-type EHA105. In summary, this study reports on the characteristics of the novel auxotrophic strains of *A. tumefaciens* along with the effective use of such auxotrophic *A. tumefaciens* strains in plant genetic transformation.

## 1. Introduction

*Agrobacterium tumefaciens* is a Gram-negative phytopathogenic bacterium that infects large numbers of plant species. During the infection process, *A. tumefaciens* transfers a segment of DNA, known as the T-DNA (“transferred DNA”), from its tumor-inducing (Ti) plasmid into the host plant cell. The T-DNA contains a series of tumor-inducing genes and integrates into the genome of the host plant, leading to the development of crown gall disease. This mechanism of infection has been exploited for obtaining stably transformed plants expressing the genes of interest. Currently, *A. tumefaciens*-mediated genetic transformation represents the most widely used method for generating genetically modified transgenic plants [1,2,3].

The process of *A. tumefaciens*-mediated genetic transformation typically involves two distinct stages as follows: the co-cultivation stage and the regeneration stage. In the co-cultivation stage, vigorously growing *A. tumefaciens* cells are required to transfer their T-DNA into plant cells. Conversely, during the regeneration stage, it is imperative to inhibit the proliferation of or even eradicate *A. tumefaciens* from the transformed plant tissue in order to facilitate the successful regeneration of transgenic seedlings with the selection of an antibiotic or herbicide. The inhibition of *A. tumefaciens* growth typically relies on the addition of high doses of antibiotics, such as carbenicillin, cefotaxime, augmentin, or timentin, to the selection medium. However, this approach not only incurs significant economic costs but also potentially compromises the regeneration efficiency of the explants. Furthermore, antibiotic control is not always successful, as *A. tumefaciens* cells tend to overgrow during the long-term regeneration stages, which impairs the explants’ ability to regenerate, and results in reduced genetic transformation efficiency [4].

To address the issue of *Agrobacterium* overgrowth, several laboratories have developed auxotrophic mutants of *A. tumefaciens*, which typically exhibit deficiencies in the biosynthesis of a specific nucleotide or amino acid, rendering the bacteria unable to thrive in plant culture media lacking that metabolite [5,6,7,8,9,10,11]. For example, the cysteine auxotroph cys-32 of *A. tumefaciens* strain EHA105, generated through transposon mutagenesis, has proven effective in controlling contamination during plant co-cultivation. Likewise, methionine auxotrophs of strains LBA4404 and EHA105, developed via homologous recombination, successfully reduce bacterial overgrowth while minimizing antibiotic use, all without compromising transformation efficiency in switchgrass and rice. Additionally, the threonine auxotroph of strain AGL1 has been shown to significantly lower contamination risks. Notably, some auxotrophic strains, such as the auxotrophic *Agrobacterium* LBA4404−ThyA and the auxotrophic *Agrobacterium* ‘ATHVade, his’, are protected by patents, which may limit their accessibility and widespread use. However, these auxotrophic *A. tumefaciens* strains have been used only in limited studies [12]. To our knowledge, the impact of *A. tumefaciens* auxotrophic mutations on genetic transformation efficiency and the corresponding optimization of the conditions of mutant use have not been described in detail.

An additional advantage of using auxotrophic *A. tumefaciens* strains is the potential for the enhanced biocontainment of the genetically modified bacterium. An auxotroph requires supplementation with the corresponding metabolite to maintain normal growth, which is often unavailable in natural environments. It can be reasonably assumed that auxotrophic *A. tumefaciens* strains escaping from the laboratory would struggle to survive, thereby reducing potential biosafety risks [12]. It should be noted that experimental verification regarding the diminished adaptability of *A. tumefaciens* auxotrophs in natural environments, such as soil or plant tissues, is lacking.

In this study, we have developed novel histidine and leucine auxotrophic strains, as well as a dual-auxotrophic strain. In the current study, we have demonstrated that these strains effectively suppress the overgrowth of *A. tumefaciens* during the genetic transformation of *Nicotiana benthamiana*, and their transient expression and transformation efficiency depend on the supplementation of the strains with exogenous supplies of the corresponding amino acids (CAAs). Furthermore, we confirmed the reduced environmental adaptability of these auxotrophic strains in various plant and soil samples, indicating improved biocontainment. To facilitate access for interested researchers, we have deposited cultures of these auxotrophic *A. tumefaciens* strains to a public culture collection center, namely the China Center for Type Culture Collection.

## 2. Results

### 2.1. Generation of leuA or/and hisD Deletion Mutants

**Figure 1:**
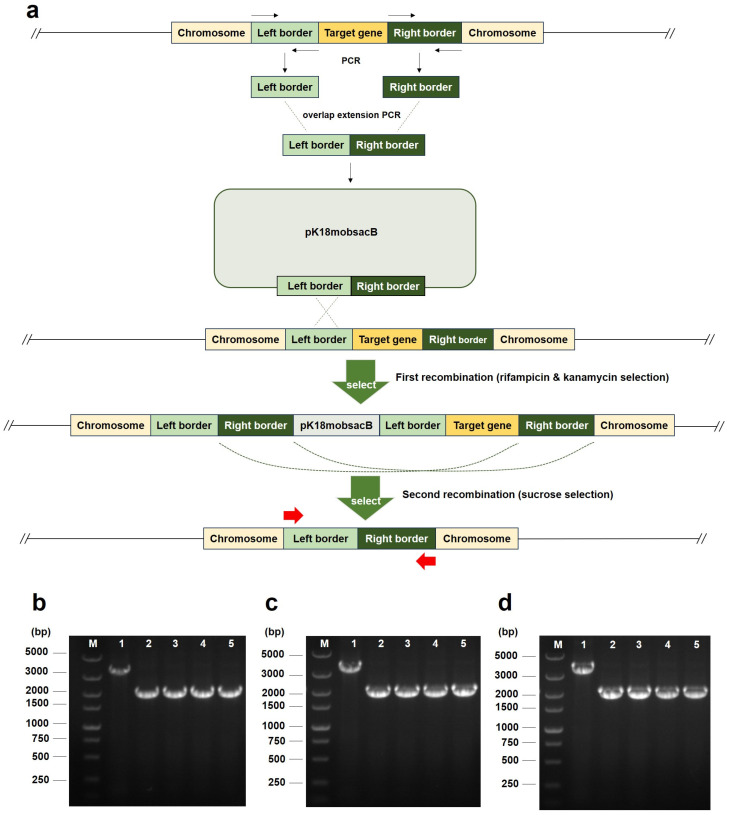
Deletion of the *hisD* or/and *leuA* genes of *Agrobacterium tumefaciens* EHA105. (**a**) Graphic illustration of target gene deletion in *Agrobacterium* by homologous crossover. The black arrows represent the primers used to amplify the left and right border of the gene. The red arrows represent the primers used to verify gene deletion. (**b**) Verification of the *hisD* deletion mutants by PCR. (**c**) Verification of the *leuA* deletion mutants by PCR. (**d**) Verification of the hisD deletion mutants in EHA105leuA− strain by PCR. Lane M, DL5000 DNA marker. Lane 1, wild-type EHA105. Lane 2–5, positive colonies following sucrose selection.

By querying the KEGG pathways, we identified histidinol dehydrogenase (*hisD*) and 2-isopropylmalate synthase (*leuA*) as key candidate enzymes involved in histidine and leucine biosynthesis, respectively, in *A. tumefaciens* C58 (Figure S1). Using a suicide plasmid carrying flanking sequences of the target genes and two rounds of homologous crossovers (Figure 1a), we successfully deleted the *hisD* and *leuA* genes individually in *A. tumefaciens* EHA105 to generate the mutant strains EHA105hisD− and EHA105leuA−, respectively (Figure 1b,c). Subsequently, the *hisD* gene was deleted in the EHA105leuA− strain to generate a double mutant strain, EHA105hisD−leuA− (Figure 1d). No additional antibiotic resistance was introduced into these deletion mutants. Furthermore, there were no significant differences observed in the growth rate of the three mutant strains on a standard LB medium compared with that of the wild-type EHA105. The cultures of the three mutants were deposited at the China Center for Type Culture Collection under accession numbers AB2024213 (EHA105hisD−leuA−), AB2024214 (EHA105hisD−), and AB2024215 (EHA105leuA−).

### 2.2. Verification of Auxotrophy in Gene Deletion Mutants

**Figure 2:**
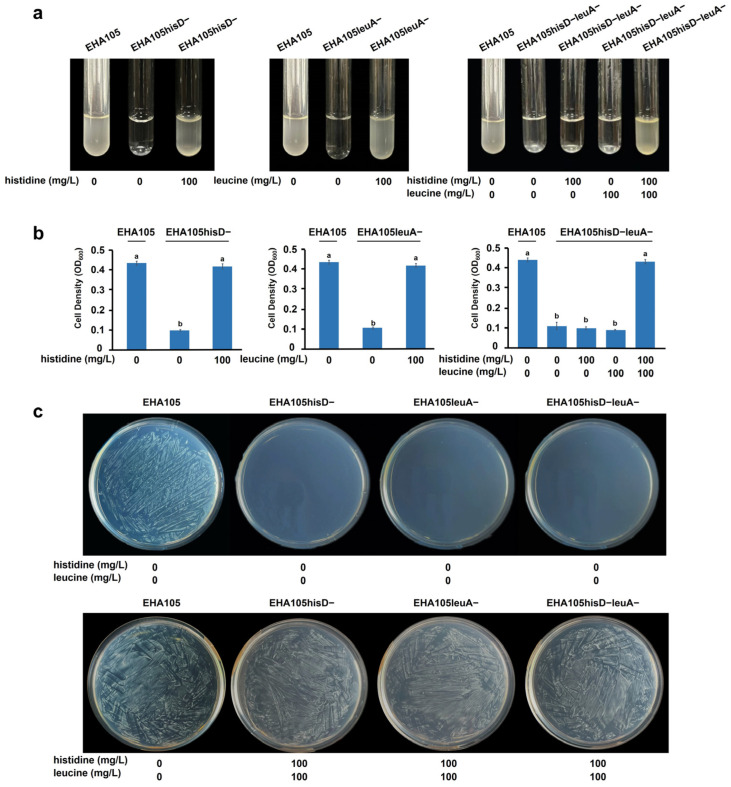
Verification of the auxotrophic phenotype of *Agrobacterium tumefaciens* gene deletion mutants. (**a**) Growth of single colonies of wild-type EHA105, EHA105hisD−, EHA105leuA−, and EHA105hisD−leuA− in liquid AB minimal medium. (**b**) Optical densities of cells of wild-type *Agrobacterium* EHA105 and auxotrophic strains in liquid AB minimal medium. Each initial cell density was OD_600_ = 0.1. Different letters in the bar charts indicate statistically significant differences calculated using one-way analysis of variance followed by Tukey’s test (*p* < 0.05). Bars indicate the mean ± standard error (SE) (from three independent experiments). (**c**) Growth of wild-type EHA105 and gene deletion mutants on Murashige and Skoog (MS) medium plates. An aliquot of 50 μL suspension (OD_600_ = 0.1) was spread onto each plate. The images and cell density values were recorded 2 d post-infiltration (dpi) at 28 °C.

To investigate the impact of *leuA* and *hisD* deletions on amino acid requirements in *A. tumefaciens*, we assessed the growth rates of the EHA105hisD−, EHA105leuA−, and EHA105hisD−leuA− strains on AB minimal medium that consisted of a limited number of inorganic salts. Following inoculation with single colonies in liquid AB minimal medium for 2 days, no visible growth was observed for EHA105hisD−, EHA105leuA−, or EHA105hisD−leuA−. However, supplementation with 100 mg/L of the corresponding amino acids (CAAs) restored the growth of all three mutants (Figure 2a). The bacterial cultures were initiated by inoculating liquid AB minimal medium with an initial optical density at OD_600_ = 0.1. At 2 days post-inoculation (dpi), the cell density remained unchanged for the three mutants; however, upon supplementing with 100 mg/L CAAs, their cell density reached a level comparable with that of the wild-type EHA105 (Figure 2b). MS medium is commonly used in plant tissue culture and does not contain any amino acids within its formulation. After inoculation and incubation on MS plates for 2 d, a dense bacterial lawn was observed when using the wild-type EHA105; on the other hand, no visible growth was detected when employing any of the three mutant strains. Nevertheless, adding 100 mg/L CAAs to the MS medium fully rescued the growth of all three mutants (Figure 2c).

The aforementioned findings indicate that the gene deletion mutants, EHA105hisD−, EHA105leuA−, and EHA105hisD−leuA−, display complete auxotrophy, relying exclusively on histidine, leucine, or both histidine and leucine for their growth, respectively.

### 2.3. Transformation Efficiency of Auxotrophic Strains Relies on CAAs

**Figure 3:**
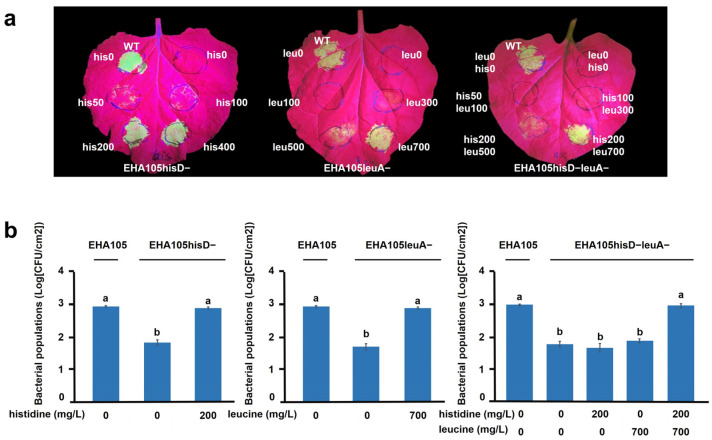
Use of the auxotrophic *Agrobacterium tumefaciens* strains for transient expression studies in *N. benthamiana*. (**a**) Detection of the enhanced green fluorescent protein (eGFP) using UV lamp excitation. The expression levels were mediated by a transformation that was performed with EHA105hisD− (left panel), EHA105leuA− (middle panel), and EHA105hisD−leuA− (right panel). Wild-type (WT) EHA105 was included in each experiment. All bacteria carried a binary vector (PH35GG) with 35S::*eGFP*. The amino acids (three-letter abbreviations) and their concentrations (0 to 700 mg/L) in the infiltration buffer were labeled at the injection sites. The GFP signal was observed at 2 days post-infiltration (dpi). (**b**) The number of surviving cells of *A. tumefaciens* at 2 dpi. Different letters above the bar charts indicate statistically significant differences, using one-way analysis of variance followed by Tukey’s test (*p* < 0.05). Bars indicate the mean ± standard error (SE) (from three independent experiments).

To evaluate the impact of the auxotrophic mutations of *A. tumefaciens* on the genetic transformation efficiency of *A. tumefaciens*, we conducted *Agrobacterium*-mediated transient assays in *Nicotiana benthamiana*, using both wild-type and auxotrophic mutant EHA105 strains carrying a binary vector (PH35GG) containing 35S::*eGFP*. Under UV lamp excitation, significantly lower transient expression levels of green fluorescence were observed for the EHA105hisD−(PH35GG), EHA105leuA−(PH35GG), and EHA105hisD−leuA−(PH35GG), compared with the wild-type EHA105(PH35GG) at 2 dpi in the absence of CAAs (Figure 3a). However, supplementation with CAAs restored the transient expression efficiency of auxotrophic strains in a concentration-dependent manner. When supplementing with the optimal CAA concentration, namely 200 mg/L histidine for EHA105hisD−(PH35GG), 700 mg/L leucine for EHA105leuA−(PH35GG), and a combination of 200 mg/L histidine and 700 mg/L leucine for EHA105hisD−leuA−(PH35GG), the green fluorescence levels were comparable with those observed in the wild-type EHA105(PH35GG) (Figure 3a). Notably, these concentrations were higher than those required for growth recovery in AB minimal medium, likely due to differences in the planta microenvironment.

We then quantified the survival of *A. tumefaciens* cells at 2 dpi. The numbers of surviving cells for EHA105hisD− (67 colony-forming units (CFU)/cm^2^, 7.18%), EHA105leuA− (56 CFU/cm^2^, 6.01%), and EHA105hisD−leuA− (45 CFU/cm^2^, 5.86%) were significantly lower than for the wild-type EHA105 (910 CFU/cm^2^, 100%) in the absence of CAAs, whereas supplementation with the optimal CAA concentration fully restored the survival rate of each mutant strain (Figure 3b).

These findings suggest that auxotrophic mutations by the deletion of *hisD* or/and *leuA* severely compromise the transient expression efficiency of *A. tumefaciens*, this being potentially attributable to a diminished reproductive or survival capacity of *A. tumefaciens* within the plant tissue. The concentrations of amino acids required for the complete restoration of transient expression efficiency varied among the different auxotrophic mutants and were markedly higher than the concentration (100 mg/L) needed for full growth restoration in artificial medium.

### 2.4. Auxotrophy Controls Overgrowth but Needs CAAs for Transformation

**Figure 4:**
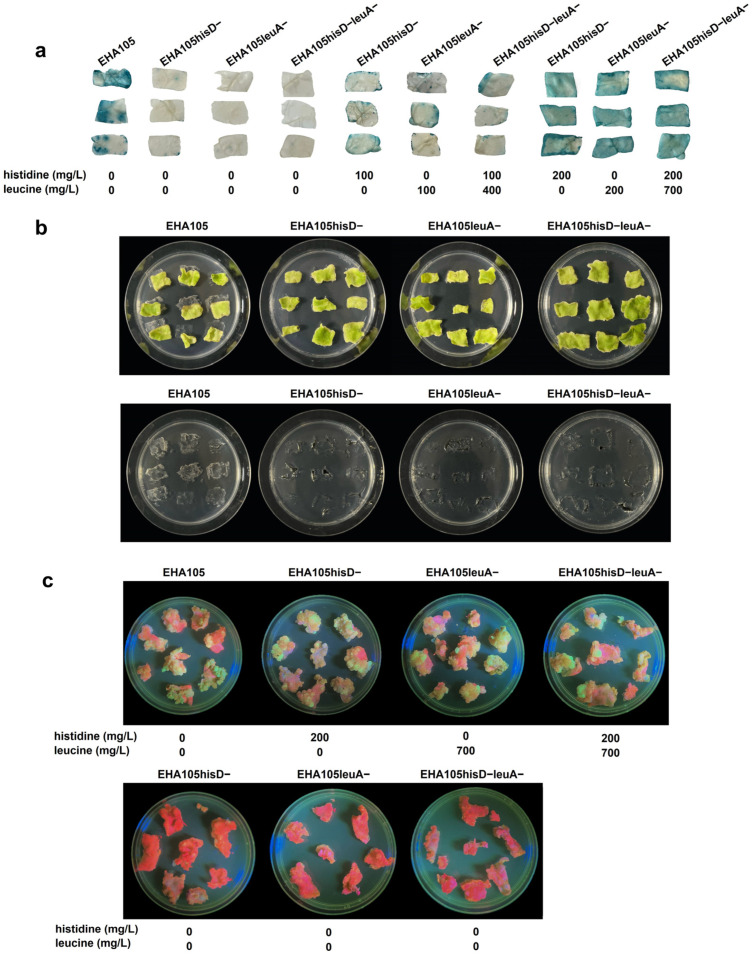
Use of auxotrophic *Agrobacterium tumefaciens* strains for the stable genetic transformation of *Nicotiana benthamiana*. (**a**) The transient GUS expression in the wild-type and auxotrophic strain-mediated transformation of *N. benthamiana* leaf disks was detected using histochemical staining. Left, without corresponding amino acids (CAAs); middle, with moderate CAA concentrations; right, with the optimal CAA concentrations in the co-cultivation medium. All the strains harbored the binary vector pCAMBIA1305, and the leaf disks were co-cultured with each strain for 2 days. (**b**) The growth of *A. tumefaciens* around (left panel) and beneath (right panel) the leaf disks. The leaf disks were incubated on the co-cultivation medium supplemented with the optimal CAA concentrations for 2 days, followed by transfer to amino acid- and antibiotic-free medium for 10 days. (**c**) Wild-type and auxotrophic strain-mediated *N. benthamiana* stable transformations with eGFP were observed using UV lamp excitation at 40 days post-infiltration (dpi).

To evaluate the ability of three auxotrophic strains to achieve stable genetic transformation, we conducted leaf disk transformation experiments on *N. benthamiana*. All strains were equipped with the binary vector pCambia1305, containing 35S::*GUS* (with a 5′-terminal intron). The standard leaf disk transformation protocol was employed without any amino acid supplementation in the co-cultivation medium and bacterial suspension. Following a 2-day co-cultivation period, GUS activity levels when using EHA105hisD−(pCambia1305), EHA105leuA−(pCambia1305), or EHA105hisD−leuA−(pCambia1305) were obviously lower than that of the wild-type EHA105(pCambia1305) (Figure 4a). However, complete restoration of GUS activity was achieved by supplementing the co-culture medium with the optimal concentrations of CAAs determined from previous eGFP transient assays on intact leaves (Figure 4a). We found that this optimal concentration used in intact leaves can also fully restore the transient expression efficiency of the mutants when applied to the leaf disk method, and this was close to that of the wild type. Moreover, when supplementation with 100 mg/L CAA in the medium was made for each amino acid, only the partial restoration of GUS activity was observed (Figure 4a).

After 2 days of co-cultivation in the presence of the optimal concentration of CAAs, the leaf disks were transferred to a regeneration medium in the absence of any amino acids or antibiotics for 10 days. As depicted in Figure 4b, abundant bacterial colonies were observed surrounding and beneath the leaf disks with the wild-type EHA105 strain, whereas no visible bacterial colonies were detected in experiments employing each of the three auxotrophic strains. Furthermore, neither EHA105leuA− nor EHA105hisD−leuA− exhibited excessive bacterial overgrowth on the leaf disk until 80 dpi in the absence of antibiotic treatment (Figure S2).

We subsequently employed EHA105(PH35GG), EHA105hisD−(PH35GG), EHA105leuA−(PH35GG), and EHA105hisD−leuA−(PH35GG) for the transformation of *N. benthamiana* leaf disks. Following a 40-day kanamycin selection period post-transformation, we observed eGFP expression in kanamycin-resistant callus. Notably, during the co-cultivation stage supplemented with the optimal concentrations of CAAs, the transformation efficiency of the three auxotrophic strains was comparable with that of the wild-type EHA105. However, without adding CAAs at the co-cultivation stage, there was a significant reduction in the production of eGFP-expressing callus using these three auxotrophic strains compared with the wild-type strain (Figure 4c).

### 2.5. Auxotrophic Mutations Reduce A. tumefaciens Survival in Natural Settings

In the aforementioned transient assay conducted in *N. benthamiana*, we observed a decreased bacterial cell survival rate at 2 dpi for the auxotrophic strains compared with the wild-type strain. Consequently, we sought to investigate whether these auxotrophic strains also exhibited a decline in fitness over an extended period or under different natural conditions.

**Figure 5:**
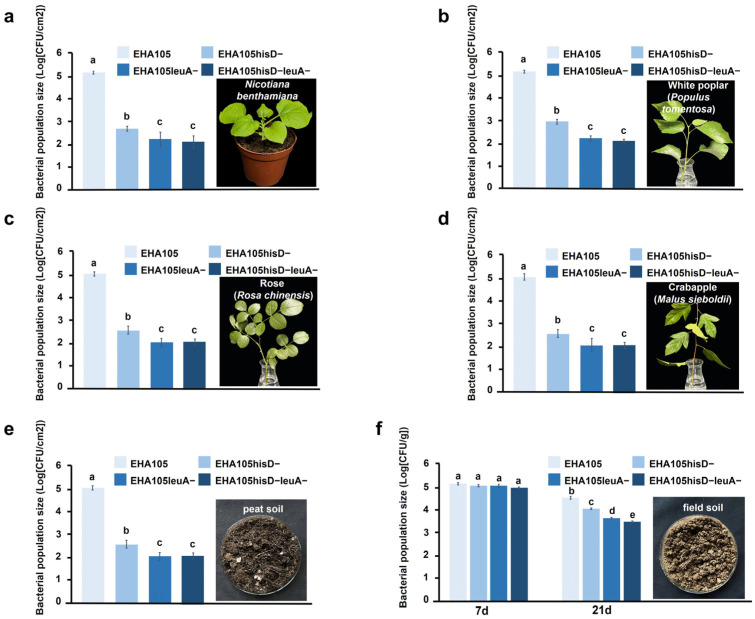
Number of surviving cells of the wild-type and auxotrophic *Agrobacterium tumefaciens* strains in leaves of *Nicotiana benthamiana* (**a**), white poplar (**b**), rose (**c**), and Toringo crabapple (**d**), and in peat soil (**e**) at 7 dpi, and field soil (**f**) at 7 and 21 dpi (days post-infiltration). Different letters in the bar charts indicate statistically significant differences calculated using a one-way analysis of variance followed by Tukey’s test (*p* < 0.05). Bars indicate the mean ± standard error, SE (from three independent experiments).

Suspensions (OD_600_ = 0.1) of the wild-type and auxotrophic strains were infiltrated into the leaves of *N. benthamiana* and detached shoots of white poplar, rose, and Toringo crabapple. At 7 dpi, the numbers of surviving cells of EHA105hisD−, EHA105leuA−, and EHA105hisD−leuA− in the leaves of these four plant species were only 0.30–0.84%, 0.11–0.30%, and 0.10–0.28%, respectively, of the values exhibited by the wild-type EHA105 (100%) (Figure 5a–d, Table S2). Subsequently, *Agrobacterium* suspensions (OD_600_ = 0.1) were homogeneously mixed in two types of sterilized soil. After incubating for seven days in commercial peat soil, the numbers of surviving cells of EHA105hisD−, EHA105leuA−, and EHA105hisD−leuA− decreased to 1.31%, 0.61%, and 0.23%, respectively, relative to the wild-type EHA105 strain (100%) (Figure 5e, Table S2). After incubation for 7 days (7 dpi) in freshly collected field soil, the surviving number of cells of the three auxotrophic strains was not significantly lower than that of the wild-type strain; however, at 21 dpi, the number of surviving cells of the three auxotrophic mutants decreased to 26.0% (EHA105hisD−), 14.9% (EHA105leuA−), and 11.4% (EHA105hisD−leuA−), respectively, relative to the wild-type EHA105 (100%) (Figure 5f, Table S2).

These findings suggest that the three auxotrophic strains exhibit a significantly diminished capacity for survival in various natural settings when compared to the wild-type strain, with the EHA105hisD−leuA− double mutant displaying the most pronounced decreases.

## 3. Discussion

### 3.1. Auxotrophic Agrobacterium Strains Generated in This Study Are Novel and Suitable for Routine Plant Genetic Manipulation

By employing various mutation induction methods, a number of auxotrophic strains of *A. tumefaciens*, dependent on the exogenous supply of different amino acids and/or nucleotides for growth, have been generated and reported [5,6,7,8,9,10,11]. One patent described a UV-light-induced mutant AGL0 strain, ‘ATHVade, his’, which is auxotrophic for adenine and histidine, but the mutated gene remains unidentified. Importantly, on minimal medium lacking histidine but containing adenine, ‘ATHVade, his’ exhibited only partial growth inhibition (approximately 58% of the optical density compared with that in exhibited the presence of both histidine and adenine) [6]. Another report described a leucine auxotrophic EHA105 strain, strain ‘leu-27’, which is a transposon insertion mutant. The specific mutated gene remains unidentified, and this mutant could not be recovered from cryopreservation [5].

In the current study, we successfully deleted key genes (*hisD* and *leuA*) involved in histidine and leucine biosynthesis, respectively, in *A. tumefaciens* EHA105, resulting in the generation of mutant strains EHA105hisD− and EHA105leuA−, as well as their double mutant strain EHA105hisD−leuA−. Our findings have demonstrated that these three mutant strains exhibit robust auxotrophic phenotypes (Figure 1a–c). Moreover, it is noteworthy that these three mutant strains lack additional antibiotic resistance, display normal growth on standard LB medium, and can be recovered from cryopreservation conditions, as demonstrated by multiple experiments preparing competent cells using glycerol stocks stored in a low-temperature freezer, making them very suitable for routine genetic manipulations.

### 3.2. Supplementation with the Corresponding Metabolites Is Crucial for Successful Genetic Transformation Using Auxotrophic A. tumefaciens Strains

The majority of media used in plant genetic transformation, such as MS, B5, NB [13], and WPM [14], do not contain nucleotides or amino acids (with only glycine present in some instances). Therefore, a valid concern arises regarding the potential limitation of the growth of auxotrophic *A. tumefaciens* strains during co-cultivation, which could potentially reduce genetic transformation efficiency. However, to our knowledge, there have been no reports addressing supplementation with the corresponding metabolites during the co-cultivation stage when using auxotrophic strains for stable genetic transformation. We have noted that, in most of the previous reports on genetic transformation or transient expression using auxotrophic *A. tumefaciens* strains, the explants used were usually callus or suspension cells [5,8,9], which may secrete the metabolites required by the auxotrophic strains, or the co-cultivation medium (mNB or N6D medium) contains casamino acid [8,9,15]. It is only seen that when using thymidine auxotrophic *A. tumefaciens* strains for the transient expression of Arabidopsis with the AGROBEST method, the corresponding thymidine was added [10].

In the present study, we observed a significant reduction in the bacterial survival rate and the impaired efficiency of transient assays (using both intact leaves and leaf disks) and the genetic transformation of *N. benthamiana*, when using the three auxotrophic EHA105 strains compared with the wild-type EHA105. However, supplementation with the appropriate concentration of CAAs restored the survival rates of the mutant strains to the wild-type levels, as well as rescuing the efficiencies of transient assays and genetic transformation (Figure 3 and Figure 4a,c). These three mutants provide more options for the optimization of various plant genetic transformations, allowing researchers to tailor their approach based on specific experimental needs and plant species. Based on the results of the transient expression experiments and genetic transformation experiments, we cautiously infer that the transformation efficiency of many auxotrophic *A. tumefaciens* strains in plant-derived explants (such as leaf disks or petiole or stem segments) may be compromised compared with the wild-type strain. This finding should be attributed to the diminished capacity of the auxotrophic strains for reproduction and survival within plant tissues. However, this impairment could be fully restored through the exogenous supply of the corresponding metabolite. Among these mutants, the double mutant exhibited the strongest dependence on the supplementation of the corresponding nutrients. The optimal metabolite concentration for supplementation could be determined through the transient expression assay using intact leaves or explants.

### 3.3. Auxotrophic A. tumefaciens Strains Are Superior for Ensuring Biocontainment in Plant Genetic Transformation

The *A. tumefaciens* strains used in plant genetic transformation carry a diverse range of target genes, as well as antibiotic- or herbicide-resistance genes for use in selection, on their T-DNA. In the natural environment, the likelihood of these genes being transferred into the germline of plants is minimal due to the relatively low efficiency of genetic transformation, despite numerous optimization efforts under laboratory conditions. Nevertheless, it remains highly desirable to prevent the introduction of these genetically modified *A. tumefaciens* strains into the natural ecosystem [12]. *A. tumefaciens* can persist for long periods in transgenic tobacco (Nicotiana tabacum) T_0_ generation, but not in the T_0_ generation seeds [16]. Due to their long juvenile period, transgenic perennial woody plants are difficult to obtain in the form of T_0_ seeds or T_1_ seedlings, whereas reports have shown that multiple rounds of culture in the presence of antibiotics are not sufficient to completely remove *A. tumefaciens* from the T_0_ generations of poplar and conifers [17,18]. In the present study, we conducted an analysis on the survival of auxotrophic EHA105 strains in *N. benthamiana*, white poplar, rose, and Toringo crabapple leaves, as well as in two sterile soil samples. Our findings demonstrated that the survival rates of the three auxotrophic strains in diverse natural environments are significantly lower than those of the wild-type strain, particularly for the double mutant EHA105hisD−leuA−, with the survival rate of the mutants ranging from 1/9 to 1/1000 of the wild-type EHA105 rate. It is important to note that these experiments represented only short-term observations (7–21 days) conducted within a controlled greenhouse or sterile environment. Moreover, it would be anticipated that these auxotrophic strains would face even greater challenges when competing with other microorganisms in natural open non-sterile environments over an extended period. Therefore, the utilization of these auxotrophic strains could effectively minimize the escape and establishment of genetically modified *A. tumefaciens* into the environment. Furthermore, considering that EHA105hisD−leuA− involves mutations in two different amino acid biosynthesis pathways, the likelihood of spontaneous reverse mutation is significantly diminished, thereby providing an enhanced biocontainment option for plant genetic modification.

## 4. Materials and Methods

### 4.1. Gene Deletion and Verification of the Phenotypes of A. tumefaciens Auxotrophic Mutants

According to the amino acid biosynthesis pathways of *A. tumefaciens* C58 from the Kyoto Encyclopedia of Genes and Genomes (KEGG) database, the key genes involved in the biosynthesis of histidine and leucine were selected for gene deletion (Figure S1). The gene deletion experiments of *A. tumefaciens* are based on a protocol for generating mutants of *Pseudomonas syringae* pv. tomato DC3000 [19], with minor modifications (Figure 1a). In brief, the flanking sequences approximately 1000 bp upstream and downstream of the target genes were amplified with Phanta Flash Master Mix (Vazyme, Nanjing, China) (Table S1). Then, using the ClonExpress Ultra One-Step Cloning Kit (Vazyme, Nanjing, China), the upstream and downstream sequences were seamlessly assembled together and recombined into the *Eco*RI and *Sal*I double-digested site of the pK18mobsacB (kanamycin resistance) plasmid. The recombined pK18mobsacB plasmids were electroporated into *Escherichia coli* S17-1, and then, the plasmids were transformed from S17-1 to *A. tumefaciens* EHA105 (rifampicin resistance) by biparental mating. The mating mixture was selected on LB (Luria–Bertani, Sangon Biotech, Shanghai, China) plates supplemented with rifampicin (100 mg/L) and kanamycin (50 mg/L). Because the colE1 ori of pK18mobsacB plasmids could not support replication in *A. tumefaciens*, the positive colonies grown on rifampicin and kanamycin plates were EHA105 strains, with pK18mobsacB integrated into their chromosomes. Subsequently, positive colonies were counter-selected on LB plates containing sucrose (10%, *w*/*v*) to obtain EHA105 clones with the target gene and pK18mobsacB backbone deleted by secondary crossover. PCR was used to verify the deletion of the target genes. The auxotrophic phenotypes were verified through growth experiments in liquid AB minimal medium [20] and solid Murashige and Skoog (MS) medium [21], with or without the corresponding amino acids (CAAs).

### 4.2. Transient Assay in N. benthamiana and GUS Staining of Leaf Disks

*N. benthamiana* seedlings were grown in a greenhouse at 25 °C with a 12 h light/12 h dark photoperiod and 60% relative humidity. *A. tumefaciens* strains carrying the binary vector PH35GG, which contains 35S::*eGFP* and the kanamycin-resistant gene on T-DNA, were first streaked onto LB medium supplemented with rifampicin (100 mg/L) and kanamycin (50 mg/L) and incubated at 28 °C for 48 h to obtain single colonies. A single colony was then inoculated into liquid LB medium with rifampicin (100 mg/L) and kanamycin (50 mg/L) and grown overnight at 28 °C with shaking at 200 rpm. The bacterial cells were harvested by centrifugation, washed, and resuspended and activated in a liquid ¼-strength MS (pH 5.7) medium containing 150 mM acetosyringone. The suspensions were adjusted to a final concentration of OD_600_ = 0.1, supplemented with the corresponding concentration of CAAs, and they were then infiltrated into leaves of 4-week-old seedlings of *N. benthamiana*. The enhanced green fluorescent protein (eGFP) signal was detected using a portable UV lamp (LUYOR-3415, Shanghai, China) at 2 d post-infiltration (dpi).

The GUS staining of leaf disks was performed using the GUS Staining Kit (Huayueyang, Beijing, China) at 2 d post-infiltration (dpi). After staining, the leaf disks were then decolorized 3–5 times, using 50% and 100% alcohol.

### 4.3. Quantification of the Number of Surviving Cells of A. tumefaciens in Various Plant and Soil Environments

The current season’s shoots (with leaves), approximately 40 cm in length, from Toringo crabapple (*Malus sieboldii*), rose (*Rosa chinensis*), and white poplar (*Populus tomentosa*), were collected in June on the campus of Nanjing Forestry University. In order to detect surviving *A. tumefaciens* cells in different plant samples, a bacterial suspension in 10 mM MgCl_2_ (OD_600_ = 0.1) was infiltrated into the leaves of 4-week-old seedlings of *N. benthamiana* using a needle-less syringe, and the shoots of crabapple, rose, and poplar were subjected to vacuum infiltration for 1 min with the bacterial suspension containing 0.01% (*v*/*v*) Silwet L-77. After removing the surface liquid with a tissue, the cut shoot ends were immersed in water and placed in a greenhouse maintained at 60% relative humidity and 25 °C under a 12 h light/12 h dark photoperiod. After an incubation period of 7 d, the leaves were sterilized with 5% (*v/v*) hydrogen peroxide for 3 min. The 1-cm^2^ leaf disks were excised and subsequently ground with silica sand in an aliquot (1 mL) of 10 mM MgCl_2_. The resulting mixture was then diluted 10- or 100-fold, and it was then spread on LB plates (containing rifampicin, 100 mg/L). To quantify the number of surviving cells in various soil samples, 1 mL of the bacterial suspension with an OD_600_ = 0.1 was evenly mixed in 50 g sterile soil. Then, the soil samples were wrapped in plastic wrap and incubated at 28 °C. At 7 dpi, a 1 g soil sample was resuspended in 1 mL of a 10 mM MgCl_2_ solution. After the soil particles were allowed to naturally settle out, the supernatant was diluted 10- or 100-fold and spread on LB plates (containing rifampicin, 100 mg/L). The resulting colonies were counted and subjected to statistical analysis based on three independent experiments.

### 4.4. Stable Genetic Transformation of N. benthamiana

The stable genetic transformation of *N. benthamiana* was performed according to the method described by Horsch et al. with minor modifications [22]. Briefly, leaf disks excised from 4-week-old aseptic *N. benthamiana* seedlings were inoculated with *A. tumefaciens* suspension (OD_600_= 0.5, in ¼-strength liquid MS medium) for approximately 10 min. Co-cultivation was carried out on regeneration medium (MS medium supplemented with 0.1 mg/L naphthalene-acetic acid and 1 mg/L 6-benzyladenine) for 2 d, and then, the leaf disks were transferred to the regeneration medium supplemented with 50 mg/L kanamycin and 200 mg/L timentin.

## Data Availability

The data supporting the findings of this study are provided in the main text and Supplementary Materials.

## Supplementary Materials

The data are provided in the document. The following supporting information can be downloaded at: https://www.mdpi.com/article/10.3390/plants14060925/s1, Figure S1: KEGG pathway map of histidine (a) and leucine (b) biosynthesis in *A. tumefaciens* C58; Figure S2: Leaf disks of *N. benthamiana* co-cultivated with EHA105leuA− (left) and EHA105hisD−leuA− (right) at 80 dpi. The leaf disks were co-cultivated with auxotrophic strains in the presence of optimal CAA concentrations for 2 days, followed by transfer to a regeneration medium lacking amino acids and antibiotics; Table S1: Primers used in this study; Table S2: Quantification of surviving cells of the wild-type and auxotrophic strains in various natural settings.
