# Supplementary material for: Effective Use of Novel Auxotrophic Agrobacterium tumefaciens Strains for Transformation and Biocontainment
Source: Plants (Basel). 2025 Mar 15;14(6):925. doi: 10.3390/plants14060925 (PMC11944849; doi:10.3390/plants14060925)
Supplement: Supplementary file 1 [file plants-14-00925-s001.zip › plants-3516997-supplementary.pdf]

Table S1 Primers used in this study

Primer name	Forward primer and reverse primers (5′ to 3′)	Note
hisD-UP	CTTGCATGCCTGCAGGTCGACCAGTACCGATTTTCCCGATGTAA	Amplify the upstream 1,000 bp fragment of the EHA105 *hisD* gene
	TGAAAGGGTAATGGCGCGATGGCTTCAGGCGAT	
hisD-DN	ATCGCGCCATTACCCTTTCATTGTCCC	Amplify the downstream 1,000 bp fragment of the EHA105 *hisD* gene
	TATGACCATGATTACGAATTCGCGTTCGCAGGGCGTCTC	
leuA-UP	CTTGCATGCCTGCAGGTCGACTCGAGACCAAATCACCGGAT	Amplify the upstream 1,000 bp fragment of the EHA105 *leuA* gene
	AGGACGCGACGAAGCCTCAAGGGTGGGGT	
leuA-DN	TTGAGGCTTCGTCGCGTCCTCCTTGCGC	Amplify the downstream 1,000 bp fragment of the EHA105 *leuA* gene
	TATGACCATGATTACGAATTCCTTCGGTGTCGATATTCCCG	

Table S2 The quantification of surviving cells of the wild-type and auxotrophic strains in various natural settings

Natural settings	Days post inoculation	Strains	CFU/cm^2 (g)
tobacco	7	wild-type	155000
tobacco	7	EHA105hisD-	766
tobacco	7	EHA105leuA-	233
tobacco	7	EHA105hisD- EHA105leuA-	166
white poplar	7	wild-type	106333
white poplar	7	EHA105hisD-	900
white poplar	7	EHA105leuA-	316
white poplar	7	EHA105hisD- EHA105leuA-	300
rose	7	wild-type	117000
rose	7	EHA105hisD-	400
rose	7	EHA105leuA-	133
rose	7	EHA105hisD- EHA105leuA-	133
crabapple	7	wild-type	135333
crabapple	7	EHA105hisD-	400
crabapple	7	EHA105leuA-	166
crabapple	7	EHA105hisD- EHA105leuA-	133
peat soil	7	wild-type	71400
peat soil	7	EHA105hisD-	933
peat soil	7	EHA105leuA-	433
peat soil	7	EHA105hisD- EHA105leuA-	166

field soil	7	wild-type	166667
field soil	7	EHA105hisD-	154667
field soil	7	EHA105leuA-	154000
field soil	7	EHA105hisD- EHA105leuA-	139333
field soil	21	wild-type	58000
field soil	21	EHA105hisD-	16130
field soil	21	EHA105leuA-	7786
field soil	21	EHA105hisD- EHA105leuA-	6673

A

B

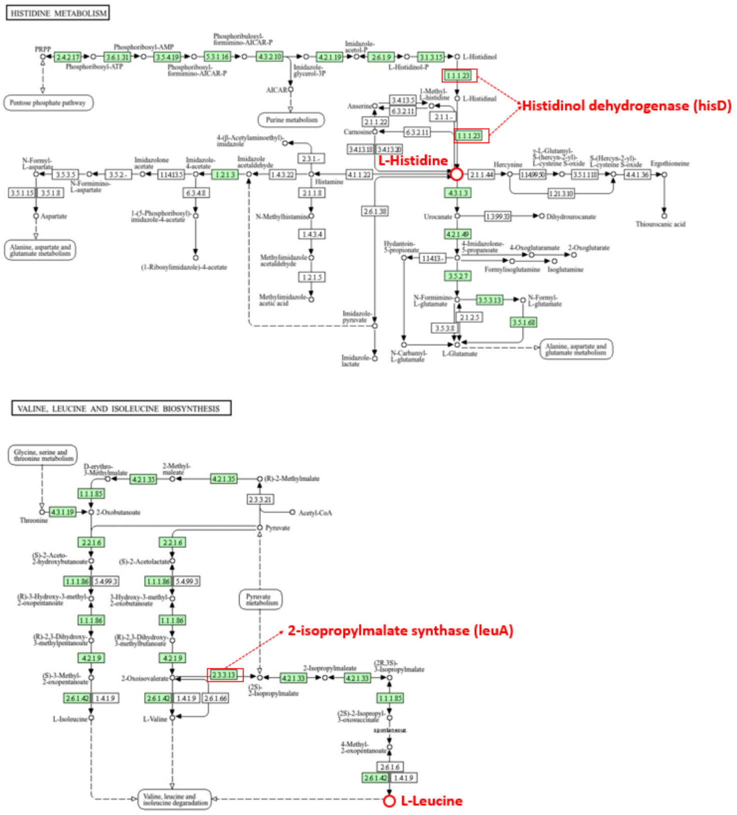

Figure S1 KEGG Pathway map of histidine (A) and leucine (B) biosynthesis in *A. tumefaciens* C58.

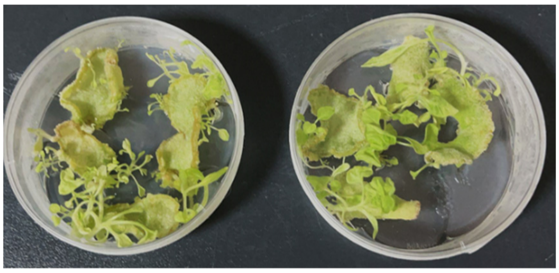

Figure S2 The leaf discs of *N. benthamiana* co-cultivated with EHA105leuA- (left) and EHA105hisD-leuA- (right) at 80 dpi. The leaf discs were co-cultivated with auxotrophic strains in the presence of optimal CAA concentrations for 2 days, followed by transfer to a regeneration medium lacking amino acids and antibiotics.
